# Phenotypic antimicrobial resistance patterns of *Escherichia coli* isolates from healthy chicken and turkey flocks in Hungary differ across poultry production systems

**DOI:** 10.1016/j.psj.2026.107750

**Published:** 2026-09-10

**Authors:** Ádám Kerek, Levente Hunor Husz, Edit Szarka, Máté Hetyésy, Gergely Álmos Tornyos, Melinda Nemes-Terényi, László Kovács, Ákos Jerzsele, Csaba Varga

**Affiliations:** a Department of Pharmacology and Toxicology, University of Veterinary Medicine Budapest, István utca 2, Budapest, H-1078, Hungary; b National Laboratory of Infectious Animal Diseases, Antimicrobial Resistance, Veterinary Public Health and Food Chain Safety, University of Veterinary Medicine Budapest, István utca 2, Budapest, H-1078, Hungary; c Department of Animal Hygiene, Herd Health and Mobile Clinic, University of Veterinary Medicine, István utca 2, Budapest, H-1078, Hungary; d Department of Pathobiology, College of Veterinary Medicine, University of Illinois Urbana-Champaign, Urbana, IL, USA

**Keywords:** Escherichia coli, Antimicrobial resistance, Multidrug resistance, Turkey, Chicken

## Abstract

Antimicrobial resistance (AMR) in *Escherichia coli* from clinically healthy poultry flocks can indicate antimicrobial selection pressure and the potential entry of resistant bacteria into the food chain. Because chicken and turkey production systems differ, aggregation into a single poultry category may obscure relevant AMR heterogeneity. We characterized phenotypic AMR in 880 *E. coli* isolates collected in Hungary during 2022–2023 from breeder chickens (n = 98), broiler chickens (n = 185), egg layers (n = 127), broiler turkeys (n = 338), and turkey breeders (n = 132). Susceptibility to 11 antimicrobials representing seven class/group endpoints was determined by broth microdilution using CLSI, EUCAST, and literature-derived interpretive thresholds. Resistance profiles were summarized descriptively and by a fixed seven-cluster exploratory k-means analysis. Associations with production type and sample source were evaluated using county-random-intercept mixed-effects logistic models, with county-fixed-effect Firth logistic models used as sensitivity analyses; resistance-count burden was assessed using Conway–Maxwell–Poisson mixed-effects regression. Multidrug resistance was most frequent in broiler chickens (99.5%), followed by turkey breeders (87.1%) and broiler turkeys (84.3%), and resistance patterns differed across production categories. In resistance-count models, broiler chicken and broiler turkey isolates had higher expected numbers of resistant class/group endpoints than breeder chicken isolates (IRR = 1.63, 95% CI: 1.38–1.93; and IRR = 1.33, 95% CI: 1.20–1.47, respectively). Sensitivity analyses showed that several associations were robust in direction, whereas aminoglycoside/aminocyclitol and folate-pathway estimates were particularly model-dependent because of sparse data and limited geographic overlap. These findings support AMR surveillance stratified by poultry species and production category, while production-type associations should be interpreted within the geographic structure of the present sampling framework.

## Introduction

Antimicrobial resistance (AMR) is a One Health challenge, and commensal and pathogenic antimicrobial-resistant *Escherichia coli* can be selected, maintained, and disseminated within poultry production systems (Varga et al., 2019; Hu et al., 2022). In Europe, harmonized monitoring frameworks and integrated inter-agency analyses have shown that AMR in food-producing animals is epidemiologically relevant both in its own right and as part of the broader human-animal-food interface (ECDC et al., 2024; EFSA and ECDC, 2024). These reports have emphasized that AMR occurrence in animal bacterial populations is heterogeneous across countries, host sectors, and antimicrobial classes, underscoring the need for sector-specific surveillance rather than aggregated interpretation (European Food Safety Authority (EFSA) and European Centre for Disease Prevention and Control (ECDC), 2023; Mihailovskaya et al., 2024).

Within the AMR surveillance context, *E. coli* is useful because it is abundant in poultry production environments, easy to recover, and therefore is used as an indicator organism for AMR monitoring in food‑producing animals (Ramos et al., 2020; Sodagari and Varga, 2023). In addition, *E. coli* is also relevant as a reservoir of mobile and transferable resistance determinants that may circulate across animal, food, and environmental interfaces (Silva et al., 2023). For these reasons, indicator *E. coli* is widely used in AMR monitoring to characterize antimicrobial selection pressure and resistance patterns in food-producing animal populations (McIver et al., 2020; Pintér et al., 2023; Barnácz et al., 2024).

Large-scale and longitudinal monitoring efforts in Canada have shown that *E. coli* recovered from poultry flocks can be used to detect temporal shifts in AMR burden and to support antimicrobial stewardship policy (Varga et al., 2019; Shrestha et al., 2023). In addition, longitudinal surveillance in livestock in the Netherlands has shown that indicator *E. coli* can be used to track trends and detect changes in AMR over time (Hesp et al., 2019). At the broader European level, integrated analyses have shown that antimicrobial consumption and AMR are linked across several bacterial-host systems, although the strength and direction of these patterns were not uniform (ECDC, EFSA, and EMA, 2024). Together, these findings support the use of structured phenotypic *E. coli* datasets as epidemiological tools for AMR assessment in food-producing animal populations (European Food Safety Authority (EFSA) and European Centre for Disease Prevention and Control (ECDC), 2023; Sodagari and Varga, 2023; Farkas et al., 2024).

Within the poultry sector, chickens and turkeys are sometimes considered together despite differences in species biology and production systems. Evidence from poultry AMR surveillance across bacterial taxa indicates that resistance patterns can differ between chicken- and turkey-associated production systems, supporting the value of species- and production-specific stratification (Sodagari et al., 2024). In addition to species-related differences, variation in farm management, housing, antimicrobial-use practices, and biosecurity may influence antimicrobial selection pressure and the distribution of resistance determinants within poultry production systems (Ali et al., 2026). Consequently, aggregating chicken and turkey production systems may mask epidemiologically relevant differences in AMR patterns. Studies of poultry-associated *E. coli* show that resistance varies across sample types, production settings, and poultry categories, indicating that ‘poultry’ should not be treated as a homogeneous category (Furtula et al., 2010; Varga et al., 2019; Khong et al., 2023; Mudenda et al., 2023; Monroy et al., 2025).

These differences may have downstream public-health relevance in the food chain. Studies from slaughter plant samples and retail poultry meat indicate that chicken and turkey products differ in their carriage of multidrug-resistant Enterobacteriaceae (Díaz-Jiménez et al., 2020), including extraintestinal pathogenic *E. coli* and clinically relevant resistance traits (Díaz-Jiménez et al., 2020). Poultry meat is an important source of human exposure to antimicrobial-resistant *E. coli* through the food chain, and studying AMR at the flock level remains important, even beyond a meat-safety context (Davies et al., 2024; Shoaib et al., 2025).

Poultry-associated AMR cannot be viewed solely through indicator ecology; it also reflects the clinical and production relevance of *E. coli* in birds (Varga et al., 2018). This bacterium ranges from commensal colonization to extraintestinal disease, and its AMR emergence is a concern in both veterinary and food-safety contexts. In addition, the AMR patterns can be influenced by local production practices, antimicrobial use, and population structure, highlighting the need for geographically focused and poultry production type-specific evaluation (Nhung et al., 2017; Watts and Wigley, 2024; Monroy et al., 2025).

Despite this background, directly comparable phenotypic AMR data on Hungarian chicken- and turkey-associated *E. coli* from clinically healthy flocks remain limited, particularly across multiple poultry production types within a unified analytical framework. This gap is important because poultry sector-level aggregation may obscure production-specific AMR burdens relevant to surveillance and stewardship. Therefore, the objective of this study was to characterize and compare phenotypic AMR patterns in *E. coli* isolates from clinically healthy chicken and turkey production systems in Hungary, including breeder chickens, broiler chickens, egg-layer chickens, broiler turkeys, and turkey breeders. We further aimed to (i) evaluate multidrug resistance (MDR) burden and class-level resistance profiles, (ii) assess antimicrobial class co-resistance patterns using clustering approaches, and (iii) quantify associations between resistance, poultry production type, and sample type using mixed-effects regression modeling.

## Materials and methods

### Study design and isolate set

This study was designed as a cross-sectional, isolate-based phenotypic AMR analysis of *E. coli* recovered from flocks sampled in the absence of clinical disease in large-scale poultry operations across Hungary. The analytical dataset integrated chicken- and turkey-associated isolate collections generated within the same broad sampling framework and laboratory workflow. The final *E. coli* dataset comprised 880 isolates, including 410 chicken-associated and 470 turkey-associated isolates, collected between February 7, 2022, and June 23, 2023, from 16 counties.

### Sampling framework and metadata

Farms were randomly selected within the underlying field sampling framework from large-scale commercial poultry operations. Although farm selection was random within this framework, the sampling design was not intended to provide a nationally representative prevalence estimate. During preparation of the secondary analytical dataset used for the present study, farm- and flock-level identifiers and the corresponding linkage information were permanently removed rather than retained as pseudonymous grouping codes. The analytical dataset therefore contains no farm- or flock-level code from which epidemiological grouping can be reconstructed retrospectively. County was retained as the only available geographic grouping variable. Consequently, farm- and flock-level clustering cannot be recovered or incorporated into the present statistical models. A maximum of one *E. coli* isolate per individual swab sample was included in the analytical dataset. Because multiple swabs were collected from each sampled farm, multiple isolates originating from the same farm or flock could therefore be represented in the dataset; however, no more than one isolate from any individual swab sample was included. For each farm, 15 cloacal and 15 respiratory swabs were collected according to the underlying field sampling protocol. Consequently, individual isolates could not be retrospectively linked to specific farms or flocks. Metadata recorded for each isolate included poultry species category, production type, sample type, county, flock size, and sampling date. For the present manuscript, the analysis was restricted to the variables directly relevant to the chicken-turkey comparison and the mixed-effects models. Farm-level identifiers were not available for the present analysis. Therefore, county was included as the available geographic grouping factor in the mixed-effects models to account for clustering of isolates by geographic origin.

### Bacterial isolation and storage

Samples intended for *E. coli* recovery were streaked onto ChromoBio Coliform agar. From each swab culture, one morphologically typical presumptive *E. coli* colony was selected at random from colonies displaying the characteristic morphology on ChromoBio Coliform agar and subcultured to obtain a pure culture. Species identity was subsequently confirmed by MALDI-TOF mass spectrometry (Flextra-LAB Kft., Budapest, Hungary), with mass spectra evaluated using MALDI Biotyper software version 12.0 (Bruker Daltonics GmbH, Bremen, Germany, 2024). Only isolates confirmed as *E. coli* were included in the analytical dataset. Confirmed isolates were transferred onto tryptic soy agar and preserved in Microbank cryovials at −80°C until susceptibility testing. The chicken and turkey isolate collections were generated using the same overall culture and storage workflow.

### Antimicrobial susceptibility testing

Phenotypic antimicrobial susceptibility was determined by broth microdilution following the methodology described in CLSI M07, 11th edition (Clinical and Laboratory Standards Institute, 2018). Testing was performed using in-house-prepared BRAND 96-well microtiter plates supplied by VWR International Kft. (Budapest, Hungary) and cation-adjusted Mueller–Hinton broth (CAMHB). Frozen isolates were revived in CAMHB and incubated at 35 ± 2°C for 18–24 h. Bacterial suspensions were adjusted to a 0.5 McFarland standard, appropriately diluted in CAMHB, and inoculated into wells containing twofold serial dilutions of the antimicrobial agents. Plates were incubated at 35 ± 2°C for 18–24 h, and MICs were determined visually as the lowest antimicrobial concentration showing no visible bacterial growth.

Each susceptibility-testing run included *E. coli* ATCC 25922 as the quality-control strain, together with antimicrobial-free positive growth controls and uninoculated negative sterility controls. Quality-control MICs were assessed against the applicable CLSI-recommended ranges and were within the accepted ranges in every testing run.

Antimicrobial stock solutions were prepared from analytical standards and corrected for the manufacturer-reported potency of each compound. Amoxicillin–clavulanic acid was prepared at an amoxicillin-to-clavulanic acid ratio of 2:1. Colistin sulfate was corrected for potency expressed as colistin base and dissolved in ultrapure water before preparation of the twofold dilution series.

### Antimicrobials and interpretive framework

The antimicrobial panel comprised 11 agents: amoxicillin, amoxicillin–clavulanic acid, ceftriaxone, imipenem, neomycin, spectinomycin, doxycycline, florfenicol, enrofloxacin, colistin, and trimethoprim–sulfamethoxazole. Interpretive thresholds were defined using CLSI VET01S (Clinical and Laboratory Standards Institute, 2024), CLSI M100 (Clinical and Laboratory Standards Institute, 2026), and EUCAST breakpoint tables version 16.1 (European Committee on Antimicrobial Susceptibility Testing, 2026), supplemented by literature-derived operational thresholds where no directly applicable poultry-specific *E. coli* breakpoint was available (Kerek et al., 2024; Jerzsele et al., 2025).

Resistance thresholds used in the analysis were ≥32 µg/mL for amoxicillin, ≥32/16 µg/mL for amoxicillin–clavulanic acid tested at a fixed 2:1 ratio, ≥4 µg/mL for ceftriaxone, ≥4 µg/mL for imipenem, ≥32 µg/mL for neomycin, ≥128 µg/mL for spectinomycin, ≥16 µg/mL for doxycycline, ≥16 µg/mL for florfenicol, ≥2 µg/mL for enrofloxacin, ≥4 µg/mL for colistin, and ≥4 µg/mL for the trimethoprim component of trimethoprim–sulfamethoxazole. The ceftriaxone resistance threshold of ≥4 µg/mL corresponded to the current CLSI Enterobacterales resistance breakpoint. The colistin resistance threshold was ≥4 µg/mL. Isolates below the applicable resistant threshold, including isolates within an intermediate category where defined, were classified as non-resistant for binary analyses. The antimicrobial-specific thresholds and their corresponding interpretive sources are summarized in Supplementary Table S3.

### Derived class-level endpoints and MDR definition

For downstream comparative analyses, individual compound-level resistance variables were collapsed into broader antimicrobial class endpoints as follows: aminoglycoside/aminocyclitol agents (neomycin and/or spectinomycin), amphenicols (florfenicol), beta-lactams (amoxicillin, amoxicillin-clavulanic acid, ceftriaxone, and/or imipenem), fluoroquinolones (enrofloxacin), folate pathway inhibitors (trimethoprim-sulfamethoxazole), polymyxins (colistin), and tetracyclines (doxycycline). Multidrug resistance (MDR) was operationally defined as resistance to at least one agent in at least three of the seven antimicrobial class/group endpoints evaluated in this study, following the general framework (Magiorakos et al., 2012). Because the present antimicrobial panel represented seven class/group endpoints, the resulting MDR classification reflects resistance within this specific analytical panel and should not be interpreted as directly equivalent to MDR estimates derived from broader antimicrobial panels. Neomycin and spectinomycin were combined into a single operational aminoglycoside/aminocyclitol endpoint for the comparative class-level analyses. This grouping was used to summarize resistance to the two 30S ribosomal-targeting agents included in the study panel and should not be interpreted as implying that spectinomycin is structurally an aminoglycoside or that the two agents are mechanistically identical.

### Statistical analysis

All statistical analyses were performed in R version 4.6.1 (R Foundation for Statistical Computing, Vienna, Austria) within the RStudio integrated development environment version 2026.08.0.187. Mixed-effects models, including the Conway–Maxwell–Poisson model, were fitted using the glmmTMB package (version 1.1.14). K-means clustering was performed using the base R stats package, while silhouette analysis and the Jaccard distance/Partitioning Around Medoids sensitivity analysis were performed using the cluster package (version 2.1.8.3). Figures were generated using ggplot2 (version 4.0.2), and heatmaps were constructed using ComplexHeatmap (version 2.26.0). Firth bias-reduced logistic regression sensitivity analyses were performed using the logistf package (version 1.26.1).

### Descriptive statistics

Antimicrobial resistance was encoded as a binary outcome, with resistance recorded as present (1) or absent (0) for each isolate across seven antimicrobial class/group endpoints: beta-lactams, aminoglycoside/aminocyclitol agents, tetracyclines, amphenicols, fluoroquinolones, polymyxins, and folate pathway inhibitors.

For each antimicrobial class, the proportion of resistant isolates was calculated, along with the prevalence of MDR. Corresponding 95% binomial confidence intervals were calculated using the Wilson method and reported for all estimates.

### Assessing antimicrobial class co-resistance and clustering across poultry types

Antimicrobial class co‑resistance patterns and clustering were assessed across poultry production types. A binary matrix was constructed to represent the presence (1) or absence (0) of resistance to each of the seven antimicrobial classes. These antimicrobial resistance variables were used as the sole input features for k-means clustering. Poultry production type was retained as a separate metadata variable and was not included in the clustering algorithm. Following cluster assignment, the distribution of poultry production types within each cluster was examined to aid biological interpretation of the identified resistance patterns.

As an exploratory summarization of class-level co-resistance patterns, isolates were clustered using the k-means algorithm applied to the binary antimicrobial resistance matrix. The standard k-means objective, based on squared Euclidean distances to cluster centroids, was used for the primary exploratory partitioning. Each isolate was assigned to a cluster based on its antimicrobial resistance profile. Because the primary objective of the clustering analysis was to summarize broad and interpretable class-level resistance-pattern structure rather than enumerate individual binary phenotypes, a fixed seven-cluster solution was selected for the primary exploratory analysis. The choice of k = 7 provided a parsimonious partition aligned with the seven antimicrobial class/group endpoints evaluated and yielded broadly interpretable resistance-pattern groups. This was an interpretability-driven analytical choice rather than a claim that k = 7 represented a uniquely data-derived optimum. Silhouette-based optimization across k = 2–30 was retained as a sensitivity analysis and is presented in the Supplementary Material.

As an additional sensitivity analysis, Jaccard distance combined with Partitioning Around Medoids (PAM) was applied to the same seven binary antimicrobial class/group variables, with candidate solutions evaluated across k = 2–30. In contrast to the Euclidean k-means analysis, the Jaccard–PAM analysis showed a progressively increasing tendency toward fragmentation as k increased and did not identify a comparably distinct compact solution within the evaluated range. The dataset contained 85 unique binary antimicrobial resistance profiles, which contributed to the increasingly phenotype-specific partitioning observed with the Jaccard-based approach. The alternative analysis was therefore used as a sensitivity assessment rather than as a replacement clustering solution. The k-means clusters were interpreted as exploratory summaries of broader resistance-pattern structure rather than as discrete biological phenotypes.

The distribution of poultry production types within each cluster was summarized by calculating the proportion of isolates from each poultry category. These proportions were used to characterize poultry-type composition across resistance clusters and were summarized descriptively.

To characterize resistance patterns within clusters, cluster-level resistance signatures were summarized for each antimicrobial class. For each cluster, the number of isolates in each cluster, and the proportion of resistance was calculated across all antimicrobial classes. These cluster‑level resistance signatures were used to summarize dominant antimicrobial resistance patterns and facilitate comparisons among clusters.

Antimicrobial resistance profiles were visualized using a heatmap generated with the ComplexHeatmap package in R. The resistance matrix was displayed using a binary color scheme to distinguish resistant and non-resistant phenotypes. For visualization, hierarchical clustering was applied to isolates and antimicrobial classes, whereas k-means cluster assignments were used only to define row splits and annotate cluster membership. Additional row annotations indicated the poultry production source.

### Association between antimicrobial resistance and poultry production and sample type

Associations between antimicrobial class resistance and poultry production type and sample type were evaluated separately for each antimicrobial class using mixed‑effects logistic regression models as the primary analyses. Resistance status to a given antimicrobial class was modeled as a binary outcome (resistant = 1, non-resistant = 0). Poultry production type was included as a categorical variable with breeder chicken specified as the reference category, along with sample type (respiratory vs. cloacal samples), and these predictors were included as fixed effects. County was included as a random intercept to account for geographic clustering. Farm- or flock-level random effects could not be incorporated because the corresponding identifiers were not retained following irreversible anonymization of the original field records. Model results were expressed as adjusted odds ratios (aORs) with corresponding 95% confidence intervals (CIs), where aORs > 1 indicated increased odds of resistance relative to the reference category. Statistical significance was assessed using the Wald test, with a two-sided P < 0.05 considered statistically significant.

Model convergence was assessed for each mixed-effects logistic regression model based on the optimization output and model-generated convergence warnings. All fitted models reached numerical convergence without optimizer or singular-fit warnings. As a secondary sensitivity analysis, county-fixed-effect Firth logistic regression models were also fitted for each antimicrobial class/group outcome. Poultry production type, sample source, and county were included as fixed effects, and profile-likelihood confidence intervals were estimated. Results of the county-fixed-effect Firth sensitivity analyses for the primary predictors across all seven antimicrobial class/group endpoints, together with the additional aminoglycoside/aminocyclitol sensitivity model fitted without county, are reported in Supplementary Table S2; the complete county fixed-effect estimates are provided in sSupplementary Table S4.

### Modeling of antimicrobial resistance burden

The number of antimicrobial class/group endpoints to which each isolate was resistant was analyzed as a count outcome. A Poisson mixed-effects regression model was fitted initially, with poultry production type and sample type included as fixed effects and county included as a random intercept to account for geographic clustering. Model dispersion was assessed using the Pearson chi-square statistic divided by the residual degrees of freedom. Because diagnostic assessment indicated underdispersion, a Conway–Maxwell–Poisson mixed-effects regression model was subsequently fitted and retained for final inference. Model fit was compared using the Akaike information criterion (AIC).

Model coefficients from the final Conway–Maxwell–Poisson model were exponentiated and reported as incidence rate ratios (IRRs) with corresponding 95% confidence intervals (CIs). IRRs greater than 1 indicated a higher expected number of resistant antimicrobial classes per isolate relative to the reference category, whereas IRRs less than 1 indicated a lower expected count. Breeder chicken and cloacal sample were used as the reference categories for poultry production type and sample type, respectively. Statistical significance of fixed effects was assessed using two-sided Wald tests, with P < 0.05 considered statistically significant.

### Ethics statement

Sample collection was performed as part of routine veterinary diagnostic activities on commercial poultry farms and was not conducted as an experimental animal study. Tracheal and cloacal swabs were collected by veterinarians from clinically healthy flocks. No experimental infection, treatment, or other study-specific animal intervention was performed.

The Animal Welfare Committee of the University of Veterinary Medicine Budapest reviewed the sampling and study framework and confirmed that the work did not constitute an animal experiment under Act XXVIII of 1998 on the Protection and Humane Treatment of Animals and Decree No. 40/2013 (II. 14.) on animal experiments. Formal authorization for an animal experiment was therefore not required.

## Results

### Distribution of isolates by county and poultry production type

In total, 880 *E. coli* isolates were collected from 16 counties in Hungary, including 98 isolates from breeder chickens (11.1%), 185 from broiler chickens (21.0%), 338 from broiler turkeys (38.4%), 127 from egg layers (14.4%), and 132 from turkey breeders (15.0%). The distribution of isolates across counties and poultry production systems is summarized in Supplementary Table S1.

### Antimicrobial agent and class-level resistance by poultry production type

Antimicrobial agent-level resistance patterns differed substantially across poultry production types (Table 1; Supplementary Fig. S1). Antimicrobials in the beta-lactam class exhibited heterogeneous resistance profiles across poultry production types. Amoxicillin resistance was the most common beta-lactam phenotype, ranging from 28.6% in breeder chickens to 76.2% in broiler chickens, with elevated prevalence also observed in broiler turkeys (63.9%) and turkey breeder isolates (55.3%). Resistance to amoxicillin-clavulanic acid was lower but varied considerably among production types, reaching 37.3% in broiler chickens compared with only 1.0% in breeder chickens. Ceftriaxone resistance showed a distinct pattern, with the highest prevalence in breeder chickens (38.8%), compared with 16.2% in broiler chickens, 28.1% in broiler turkeys, 23.6% in egg layers, and 13.6% in turkey breeders.

**Table 1:** Proportion of antimicrobial agent-specific resistance among *Escherichia coli* isolates from different poultry production types in Hungary. Values are presented as n/N (%) with 95% confidence intervals (CI)^a^.

Poultry type	AMC	AMX	COL	CRO	DOX	ENR	FFC	IMP	NEO	SPT	SXT
Breeder chicken (n=98)	1/98 (1.0%; 0.2–5.6)	28/98 (28.6%; 20.6–38.2)	38/98 (38.8%; 29.7–48.7)	38/98 (38.8%; 29.7–48.7)	48/98 (49.0%; 39.3–58.7)	47/98 (48.0%; 38.3–57.7)	21/98 (21.4%; 14.5–30.5)	2/98 (2.0%; 0.6–7.1)	34/98 (34.7%; 26.0–44.5)	27/98 (27.6%; 19.7–37.1)	90/98 (91.8%; 84.7–95.8)
Broiler chicken (n=185)	69/185 (37.3%; 30.7–44.5)	141/185 (76.2%; 69.6–81.8)	55/185 (29.7%; 23.6–36.7)	30/185 (16.2%; 11.6–22.2)	93/185 (50.3%; 43.1–57.4)	124/185 (67.0%; 60.0–73.4)	159/185 (85.9%; 80.2–90.2)	40/185 (21.6%; 16.3–28.1)	135/185 (73.0%; 66.2–78.9)	84/185 (45.4%; 38.4–52.6)	161/185 (87.0%; 81.4–91.1)
Broiler turkey (n=338)	63/338 (18.6%; 14.8–23.1)	216/338 (63.9%; 58.7–68.8)	118/338 (34.9%; 30.0–40.1)	95/338 (28.1%; 23.6–33.1)	216/338 (63.9%; 58.7–68.8)	217/338 (64.2%; 59.0–69.1)	221/338 (65.4%; 60.2–70.3)	44/338 (13.0%; 9.8–17.0)	226/338 (66.9%; 61.7–71.7)	180/338 (53.3%; 47.9–58.5)	287/338 (84.9%; 80.7–88.3)
Egg layer (n=127)	21/127 (16.5%; 11.1–24.0)	61/127 (48.0%; 39.5–56.7)	22/127 (17.3%; 11.7–24.8)	30/127 (23.6%; 17.1–31.7)	42/127 (33.1%; 25.5–41.6)	46/127 (36.2%; 28.4–44.9)	77/127 (60.6%; 51.9–68.7)	19/127 (15.0%; 9.8–22.2)	82/127 (64.6%; 55.9–72.3)	25/127 (19.7%; 13.7–27.4)	95/127 (74.8%; 66.6–81.5)
Turkey breeder (n=132)	24/132 (18.2%; 12.5–25.6)	73/132 (55.3%; 46.8–63.5)	12/132 (9.1%; 5.3–15.2)	18/132 (13.6%; 8.8–20.5)	81/132 (61.4%; 52.8–69.2)	78/132 (59.1%; 50.6–67.1)	111/132 (84.1%; 76.9–89.4)	4/132 (3.0%; 1.2–7.5)	79/132 (59.8%; 51.3–67.8)	41/132 (31.1%; 23.8–39.4)	107/132 (81.1%; 73.5–86.8)

a AMC = amoxicillin-clavulanic acid; AMX = amoxicillin; COL = colistin; CRO = ceftriaxone; DOX = doxycycline; ENR = enrofloxacin; FFC = florfenicol; IMP = imipenem; NEO = neomycin; SPT = spectinomycin; SXT = trimethoprim-sulfamethoxazole.

Resistance to folate pathway inhibitors was consistently high across all categories, whereas resistance to other classes showed marked production-type variation (Table 2, Fig. 1). Broiler chicken isolates showed the highest overall resistance burden across multiple classes, particularly beta-lactams, amphenicols, aminoglycoside/aminocyclitol, and folate pathway inhibitors, whereas turkey breeder isolates were notable for high amphenicol resistance but low polymyxin resistance.

**Table 2:** Multidrug resistance (MDR) and antimicrobial class-specific resistance among *Escherichia coli* isolates from different poultry production types in Hungary. Values are presented as n/N (% resistant; 95% confidence interval)^a^.

Poultry type	MDR	AG	AP	BL	FQ	FPI	PM	TC
Breeder chicken	57/98 (58.2%; 48.3–67.4)	42/98 (42.9%; 33.5–52.7)	21/98 (21.4%; 14.5–30.5)	56/98 (57.1%; 47.3–66.5)	47/98 (48.0%; 38.3–57.7)	90/98 (91.8%; 84.7–95.8)	38/98 (38.8%; 29.7–48.7)	48/98 (49.0%; 39.3–58.7)
Broiler chicken	184/185 (99.5%; 97.0–99.9)	155/185 (83.8%; 77.8–88.4)	159/185 (85.9%; 80.2–90.2)	164/185 (88.6%; 83.3–92.5)	124/185 (67.0%; 60.0–73.4)	161/185 (87.0%; 81.4–91.1)	55/185 (29.7%; 23.6–36.7)	93/185 (50.3%; 43.1–57.4)
Broiler turkey	285/338 (84.3%; 80.1–87.8)	262/338 (77.5%; 72.8–81.6)	221/338 (65.4%; 60.2–70.3)	259/338 (76.6%; 71.8–80.8)	217/338 (64.2%; 59.0–69.1)	287/338 (84.9%; 80.7–88.3)	118/338 (34.9%; 30.0–40.1)	216/338 (63.9%; 58.7–68.8)
Egg layer	87/127 (68.5%; 60.0–75.9)	86/127 (67.7%; 59.2–75.2)	77/127 (60.6%; 51.9–68.7)	73/127 (57.5%; 48.8–65.7)	46/127 (36.2%; 28.4–44.9)	95/127 (74.8%; 66.6–81.5)	22/127 (17.3%; 11.7–24.8)	42/127 (33.1%; 25.5–41.6)
Turkey breeder	115/132 (87.1%; 80.3–91.8)	85/132 (64.4%; 55.9–72.0)	111/132 (84.1%; 76.9–89.4)	80/132 (60.6%; 52.1–68.5)	78/132 (59.1%; 50.6–67.1)	107/132 (81.1%; 73.5–86.8)	12/132 (9.1%; 5.3–15.2)	81/132 (61.4%; 52.8–69.2)

a **MDR** (multidrug resistance) was defined as resistance to ≥3 antimicrobial class/group endpoints. **AG** = aminoglycoside/aminocyclitol agents; **AP** = amphenicols; **BL** = beta-lactams; **FQ** = fluoroquinolones; **FPI** = folate pathway inhibitors; **PM** = polymyxins; **TC** = tetracyclines.

**Fig. 1:**
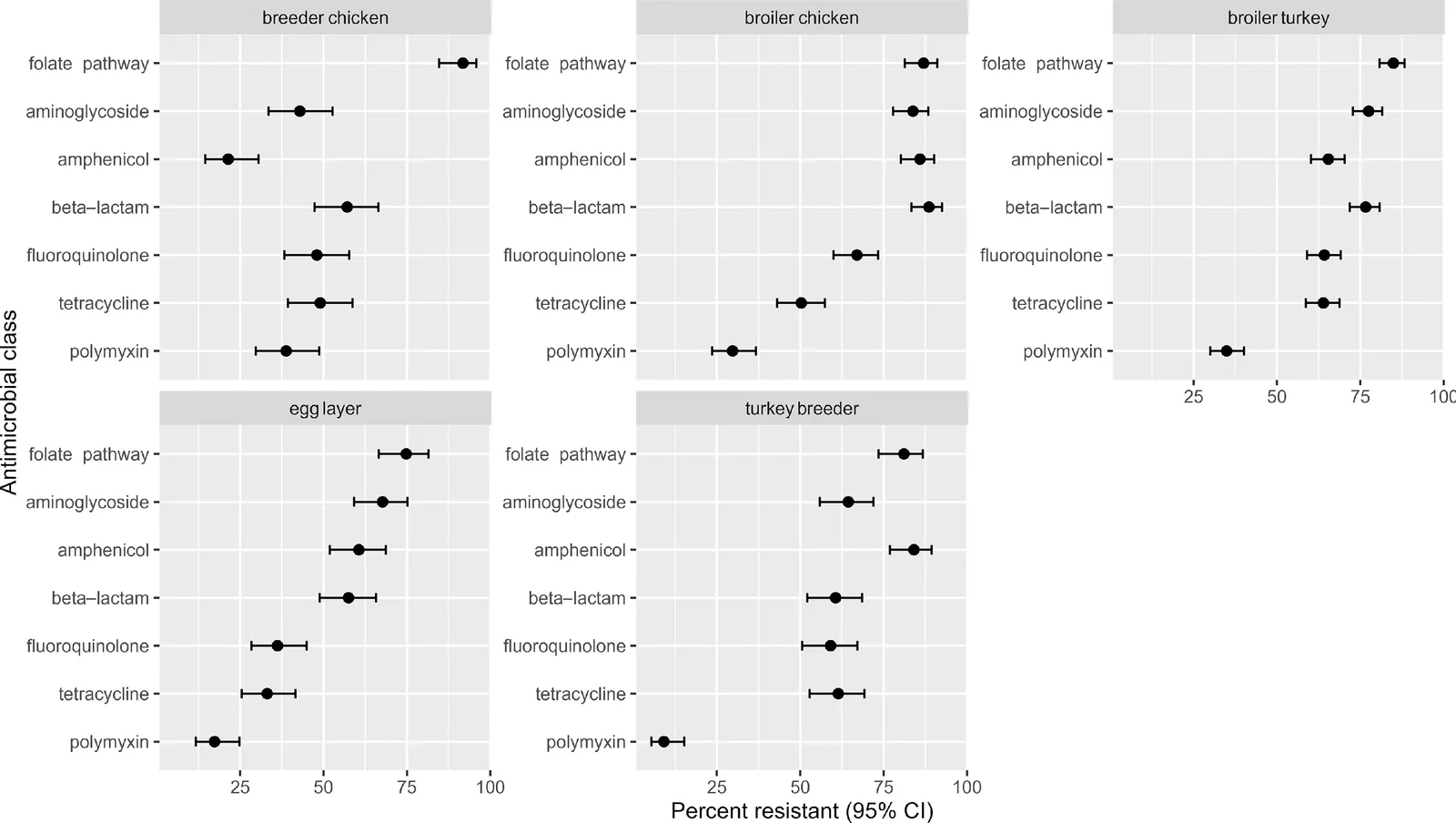
Antimicrobial class/group-level resistance among *Escherichia coli* isolates across poultry production types in Hungary. Points represent the percentage of resistant isolates and horizontal bars indicate 95% Wilson binomial confidence intervals. Panels represent breeder chicken, broiler chicken, broiler turkey, egg-layer, and turkey breeder isolates.

Among *E. coli* isolates from breeder chickens, resistance was highest to folate pathway inhibitors (91.8%, 95% CI: 84.7–95.8). Moderate levels of resistance were observed to beta‑lactams (57.1%) and tetracyclines (49.0%), as well as fluoroquinolones (48.0%). Resistance to aminoglycoside/ aminocyclitol was observed in 42.9% of isolates, while polymyxin resistance remained lower (39.8%). Resistance to amphenicols was least frequent in this production type (21.4%).

Isolates from broiler chickens exhibited the highest overall resistance levels across most antimicrobial classes. Resistance for beta‑lactams (88.6%), amphenicols (85.9%), aminoglycoside/aminocyclitol endpoint (83.8%), and folate pathway inhibitors (87.0%) and fluoroquinolones (67.0%) was high. In contrast, resistance to polymyxins remained lower (30.3%, 95% CI: 24.1–37.2), and approximately half of the isolates were resistant to tetracyclines (50.3%, 95% CI: 43.1–57.4).

In broiler turkey isolates, resistance to folate pathway inhibitors was the highest (84.9%, 95% CI: 80.7–88.3). High resistance was also observed for aminoglycoside/aminocyclitol endpoint (77.5%) and beta‑lactams (76.6%). Medium resistance levels were detected for amphenicols (65.4%), tetracyclines (63.9%), and fluoroquinolones (64.2%). Polymyxin resistance was observed in just over one‑third of isolates (37.6%).

Egg‑layer isolates displayed intermediate resistance levels across most antimicrobial classes. Resistance to folate pathway inhibitors was highest (74.8%), followed by aminoglycoside/aminocyclitol endpoint (67.7%) and amphenicols (60.6%). Resistance to beta‑lactams was observed in 57.5% of isolates, while fluoroquinolone resistance was lower (36.2%). Polymyxin resistance remained low (24.4%), and one‑third of isolates were resistant to tetracyclines (33.1%).

Among turkey breeder isolates, resistance to folate pathway inhibitors was high (81.1%), and high resistance was also observed for amphenicols (84.1%). Medium resistance levels were detected for tetracyclines (61.4%), beta‑lactams (60.6%), fluoroquinolones (59.1%), and aminoglycoside/aminocyclitol endpoint (64.4%). Resistance to polymyxins was low in this production type (9.8%).

### Multidrug resistance by poultry production type

Multidrug resistance (MDR) was common across all poultry production types but varied substantially in prevalence (Table 2). Broiler chickens exhibited the highest MDR prevalence, with 99.5% of isolates classified as MDR, followed by turkey breeder isolates (87.1%) and broiler turkeys (84.3%). Lower MDR prevalence was observed among egg layers (68.5%) and breeder chickens (58.2%). These MDR proportions refer to the operational seven-endpoint panel used in the present study.

### Assessing antimicrobial class co-resistance and clustering across poultry types

To identify broad and biologically interpretable resistance-pattern groups, isolates were classified using a fixed seven-cluster exploratory k-means solution based on the seven antimicrobial resistance classes evaluated (Fig. 2; Table 3). Cluster sizes ranged from 69 isolates (Cluster 4) to 202 isolates (Cluster 3). The clusters differed in both antimicrobial resistance composition and poultry production type distribution.

**Fig. 2:**
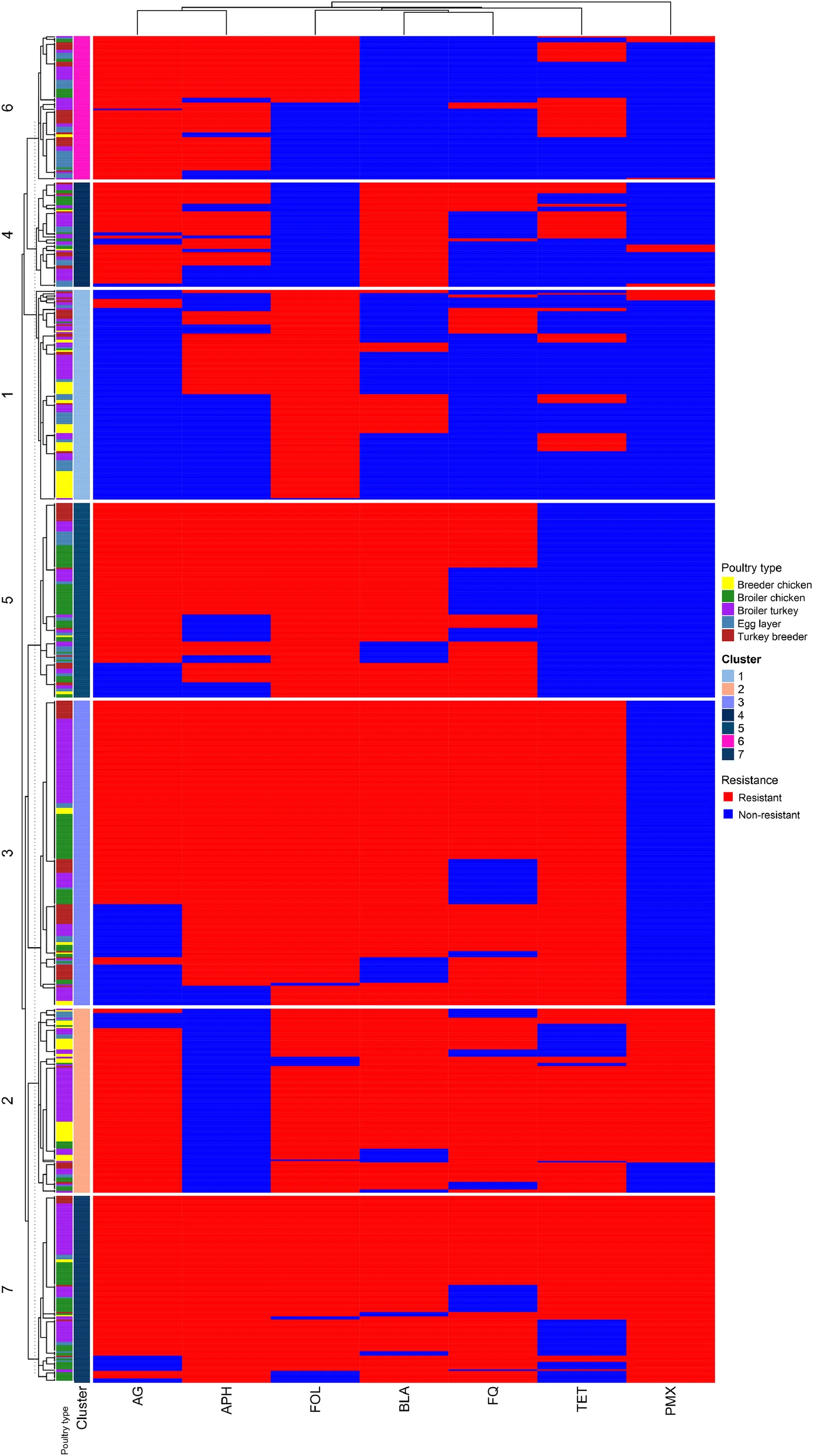
Heatmap of class/group-level antimicrobial resistance patterns among *Escherichia coli* isolates across poultry production types. Rows represent individual isolates grouped according to the fixed seven-cluster exploratory k-means solution, while columns represent the seven binary antimicrobial resistance endpoints. Red indicates resistance and blue indicates non-resistance. Poultry production type and cluster membership are shown as row annotations. AG = aminoglycoside/aminocyclitol agents; APH = amphenicols; BLA = beta-lactams; FOL = folate pathway inhibitors; FQ = fluoroquinolones; PMX = polymyxins; TET = tetracyclines.

**Table 3:** Antimicrobial class-level resistance profiles of *Escherichia coli* clusters identified by k-means clustering and poultry production type composition of the resulting clusters. Values represent the percentage of isolates within each cluster resistant to each antimicrobial class and the percentage contribution of each poultry production type. Antimicrobial classes: BLA, beta-lactams; AG, aminoglycoside/aminocyclitol agents; TET, tetracyclines; APH, amphenicols; FOL, folate pathway inhibitors; FQ, fluoroquinolones; PMX, polymyxins.

Cluster	N	Resistance profile (proportion resistant)							Poultry type composition (proportion of isolates)				
		BLA (%)	AG (%)	TET (%)	APH (%)	FOL (%)	FQ (%)	PMX (%)	Breeder chicken (%)	Broiler chicken (%)	Broiler turkey (%)	Egg layer (%)	Turkey breeder (%)
1	139	24.5	4.3	19.4	36.7	99.3	13.7	5.0	31.7	1.4	36.0	19.4	11.5
2	122	91.0	91.8	79.5	0.0	94.3	86.9	83.6	27.9	10.7	47.5	9.0	4.9
3	202	91.6	69.3	100.0	93.6	99.0	83.2	0.0	5.0	24.8	42.1	5.0	23.3
4	69	100.0	88.4	39.1	66.7	0.0	30.4	10.1	2.9	20.3	46.4	18.8	11.6
5	129	89.1	82.2	0.0	74.4	100.0	69.0	0.0	2.3	39.5	20.9	20.9	16.3
6	95	0.0	98.9	42.1	87.4	46.3	4.2	5.3	2.1	11.6	28.4	31.6	26.3
7	124	95.2	89.5	70.2	100.0	91.9	84.7	100.0	2.4	35.5	47.6	7.3	7.3

Cluster 7 (n = 124) represented the most extensive multidrug-resistant phenotype, characterized by high resistance prevalence across all antimicrobial classes, including beta-lactams (95.2%), aminoglycoside/aminocyclitol (89.5%), tetracyclines (70.2%), amphenicols (100%), folate-pathway inhibitors (91.9%), fluoroquinolones (84.7%), and polymyxins (100%). This cluster was composed primarily of broiler chicken (35.5%) and broiler turkey (47.6%) isolates.

Cluster 3 (n = 202), the largest cluster identified, was also characterized by extensive multidrug resistance, including universal tetracycline resistance (100%) and high levels of beta-lactam (91.6%), amphenicol (93.6%), folate-pathway inhibitor (99.0%), and fluoroquinolone resistance (83.2%). Broiler turkey isolates constituted the largest proportion of this cluster (42.1%).

Cluster 5 (n = 129) showed a distinct broad-resistance profile, with high resistance to beta-lactams (89.1%), aminoglycoside/aminocyclitol agents (82.2%), amphenicols (74.4%), folate-pathway inhibitors (100%), and fluoroquinolones (69.0%), whereas tetracycline and polymyxin resistance were absent.

Cluster 2 (n = 122) similarly exhibited broad multidrug resistance, with resistance prevalences exceeding 79% for beta-lactams, aminoglycoside/aminocyclitol, tetracyclines, folate-pathway inhibitors, fluoroquinolones, and polymyxins. Nearly half of isolates in this cluster originated from broiler turkey production systems (47.5%).

In contrast, several clusters demonstrated narrower resistance profiles. Cluster 1 (n = 139) was characterized primarily by folate-pathway inhibitor resistance (99.3%) with relatively low resistance to other antimicrobial classes. Cluster 6 (n = 95) exhibited very high aminoglycoside/aminocyclitol resistance (98.9%) and high amphenicol resistance (87.4%) but limited beta-lactam and fluoroquinolone resistance. Cluster 4 (n = 69) was dominated by beta-lactam (100%), aminoglycoside/aminocyclitol resistance (88.4%), and amphenicol resistance (66.7%), while resistance to folate-pathway inhibitors was absent.

Poultry production types were distributed across multiple clusters, indicating that similar resistance phenotypes occurred in more than one production system. Nevertheless, broiler chicken and broiler turkey isolates predominated in the largest multidrug-resistant clusters (Clusters 2, 3, and 7). Egg-layer and turkey-breeder isolates were generally more dispersed among clusters and occurred less frequently in the highly multidrug-resistant groups.

As an exploratory sensitivity analysis, k-means clustering was evaluated across candidate solutions ranging from k = 2 to k = 30 using average silhouette width. The highest silhouette value occurred at k = 28 (Supplementary Fig. S2). Because the highest silhouette value occurred near the upper end of the evaluated range (k = 28 of 30), the analysis did not provide evidence of a clear interior optimum or plateau. An alternative Jaccard distance and PAM clustering approach similarly showed progressive fragmentation of resistance profiles with increasing k and did not identify a compact interior optimum. Together, these findings indicated that the exact partitioning was sensitive to clustering method and parameter selection.

The k = 28 solution produced fragmented clusters that reflected increasingly specific combinations of the seven binary antimicrobial-class resistance variables rather than broader resistance-pattern groupings. Consequently, these exploratory analyses were not used as the primary clustering framework and are provided in Supplementary Fig. S2 and Supplementary Table S5 for completeness.

Despite the high degree of cluster fragmentation, the k = 28 solution demonstrated substantial heterogeneity in resistance phenotypes among isolates. Several clusters were characterized by extensive multidrug resistance, whereas others exhibited resistance primarily to a limited subset of antimicrobial classes. Poultry production types were distributed across multiple clusters, indicating that similar resistance phenotypes occurred in more than one production system. These exploratory findings support the presence of considerable diversity in antimicrobial resistance patterns but should be interpreted cautiously given the sensitivity of the clustering solution to methodological choices.

### Associations between antimicrobial class resistance and poultry type and sample type from mixed-effects logistic regression models

The primary analyses used county-random-intercept mixed-effects logistic regression, while county-fixed-effect Firth models were fitted as sensitivity analyses (Supplementary Tables S2 and S4). Beta-lactam and polymyxin estimates, together with several amphenicol and fluoroquinolone contrasts, were directionally consistent across modeling approaches. However, selected tetracycline, amphenicol, and fluoroquinolone contrasts changed materially in magnitude or statistical classification. Aminoglycoside/aminocyclitol and folate-pathway inhibitor models showed the greatest instability, with very large and imprecise county-fixed-effect estimates. These findings indicate that the direction of several associations was robust, whereas precise effect-size interpretation was sensitive to the treatment of county for specific endpoints. The mixed-effects models were therefore retained as the primary analytical framework, with the Firth models used to assess model dependence rather than as replacement estimates. Fig. 3.

**Fig. 3:**
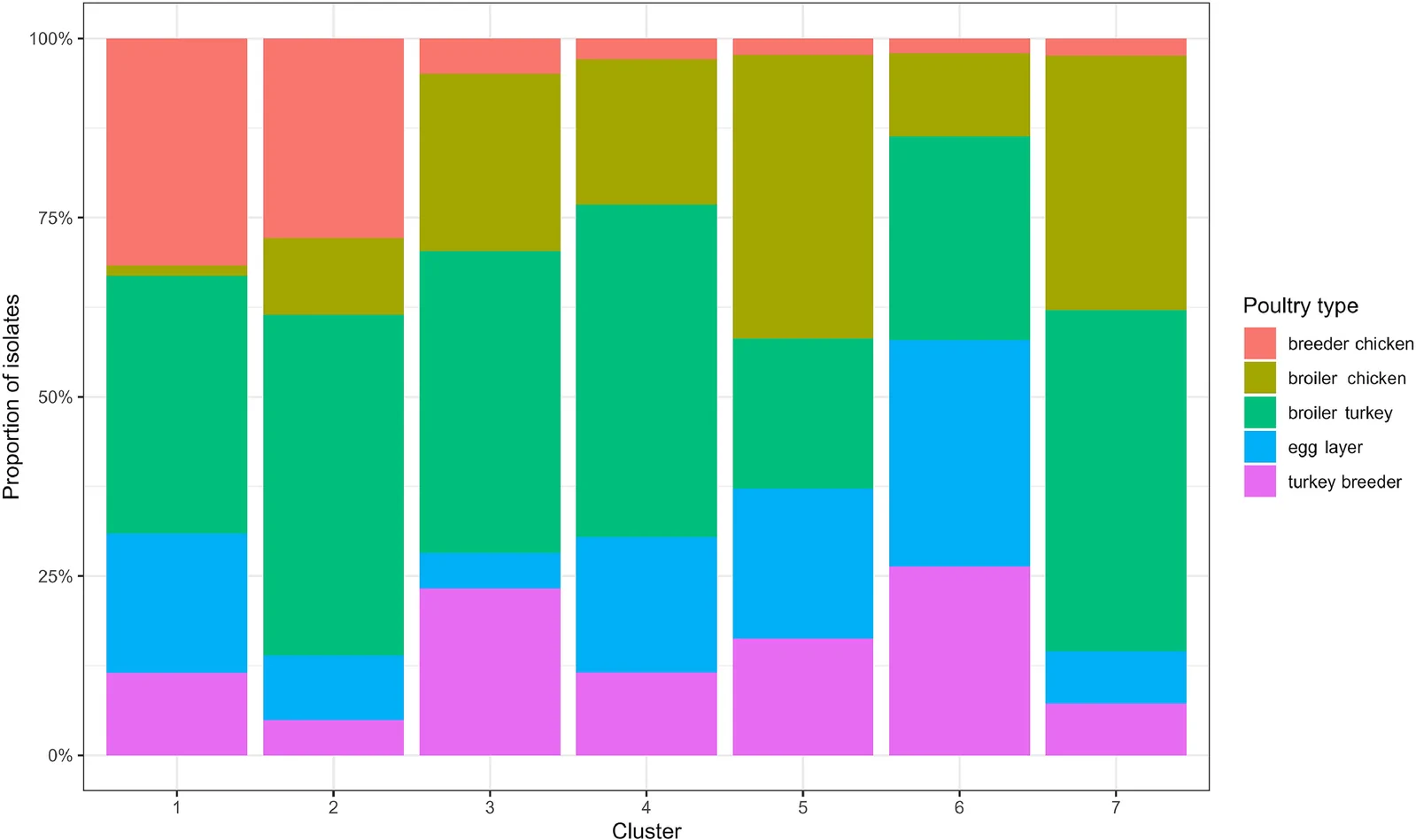
Poultry production-type composition of the fixed seven-cluster exploratory k-means solution based on class/group-level antimicrobial resistance profiles of *Escherichia coli* isolates. Bars show the proportional distribution of breeder chicken, broiler chicken, broiler turkey, egg-layer, and turkey breeder isolates within each cluster.

Mixed‑effects logistic regression models with county included as a random intercept identified significant associations between poultry production type and resistance to antimicrobial classes (Table 4; Fig. 4). All mixed-effects logistic regression models reached numerical convergence without optimizer or singular-fit warnings. However, numerical convergence did not imply stability of all effect estimates; the aminoglycoside endpoint produced exceptionally large and model-dependent estimates and was therefore evaluated separately using the bias-reduced sensitivity analyses described above.

**Table 4:** Antimicrobial class-specific resistance models for *Escherichia coli* isolates from different poultry production types in Hungary. Adjusted odds ratios (aORs) were estimated using mixed-effects logistic regression models with county as a random intercept and poultry type and sample type as fixed effects. Breeder chickens and cloacal samples were used as the reference categories. Aminoglycoside/aminocyclitol resistance is not included in this table because the county-adjusted mixed-effects model produced highly model-dependent and unstable effect-size estimates. Results of the Firth bias-reduced sensitivity analysis are provided in Supplementary Table S2.

Antimicrobial class	Predictor	aOR^a^	Lower 95% CI^b^	Upper 95% CI	P-value
Beta-lactams	Broiler chicken	4.56	1.74	11.93	0.002
	Broiler turkey	2.36	1.25	4.46	0.008
	Egg layer	0.80	0.29	2.17	0.656
	Turkey breeder	0.58	0.28	1.21	0.146
	Respiratory sample	0.93	0.67	1.29	0.667
Tetracyclines	Broiler chicken	2.04	0.84	4.95	0.117
	Broiler turkey	2.38	1.30	4.37	0.005
	Egg layer	0.60	0.25	1.46	0.259
	Turkey breeder	1.16	0.56	2.40	0.684
	Respiratory sample	1.00	0.75	1.33	0.983
	Broiler chicken	14.96	4.74	47.27	<0.001
Amphenicols	Broiler turkey	14.45	6.83	30.55	<0.001
	Egg layer	3.27	1.00	10.69	0.050
	Turkey breeder	12.44	5.41	28.62	<0.001
	Respiratory sample	0.95	0.68	1.33	0.768
Fluoroquinolones	Broiler chicken	6.34	2.13	18.85	0.001
	Broiler turkey	2.57	1.23	5.36	0.012
	Egg layer	6.57	1.93	22.40	0.003
	Turkey breeder	1.23	0.51	2.96	0.650
	Respiratory sample	1.31	0.96	1.79	0.094
Polymyxins	Broiler chicken	0.42	0.12	1.51	0.186
	Broiler turkey	0.20	0.09	0.44	<0.001
	Egg layer	0.42	0.12	1.53	0.188
	Turkey breeder	0.09	0.04	0.22	<0.001
	Respiratory sample	1.17	0.82	1.65	0.386
Folate pathway inhibitors	Broiler chicken	1.34	0.22	8.14	0.751
	Broiler turkey	0.22	0.09	0.55	0.001
	Egg layer	3.04	0.33	27.62	0.323
	Turkey breeder	4.67	0.84	25.96	0.078
	Respiratory sample	0.74	0.50	1.10	0.135

a aOR = adjusted odds ratio

b CI = confidence interval.

**Fig. 4:**
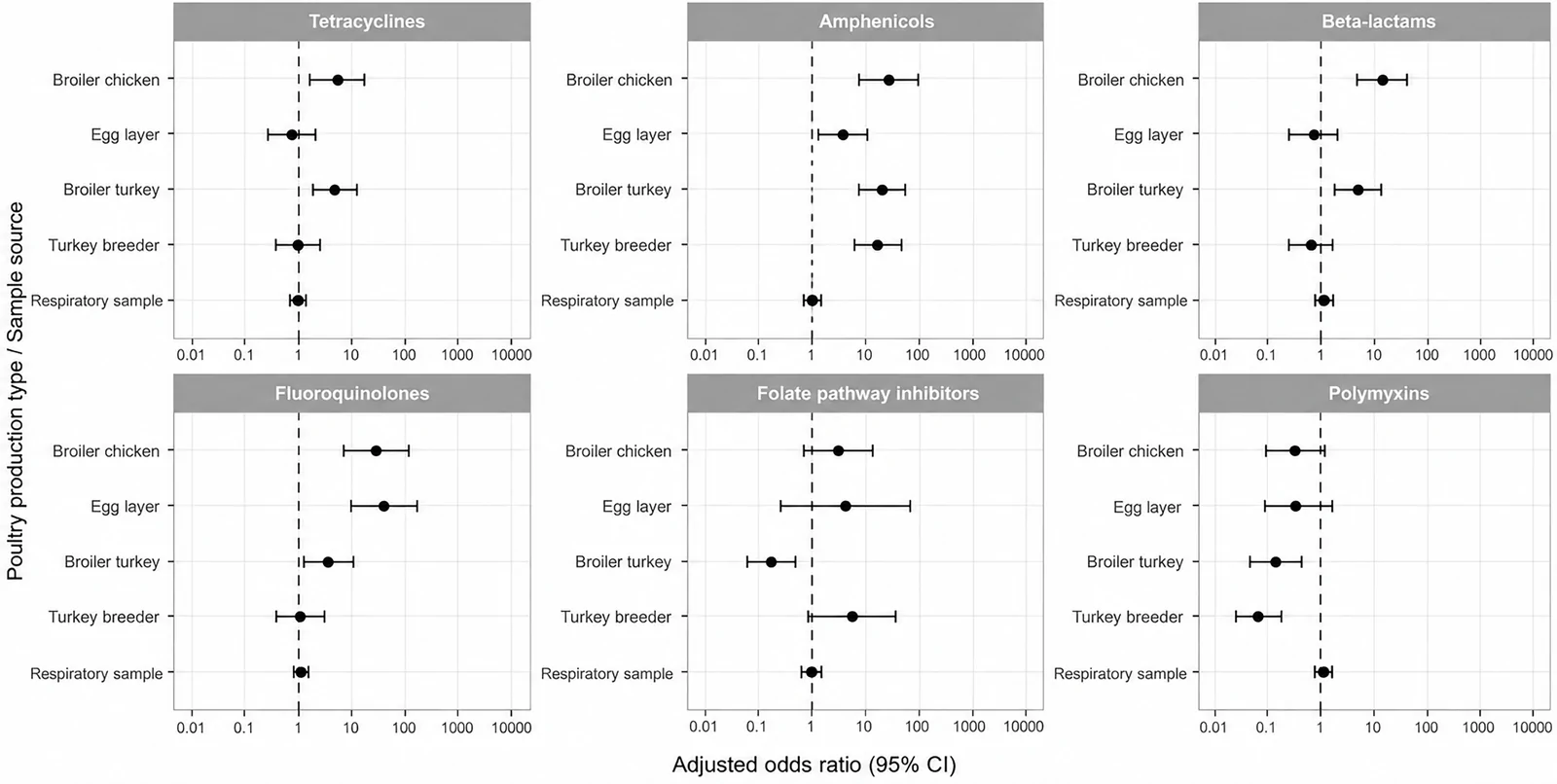
Associations between antimicrobial class/group resistance and poultry production type or sample type estimated using mixed-effects logistic regression models with county included as a random intercept. Points represent adjusted odds ratios and horizontal bars indicate 95% confidence intervals. Breeder chicken and cloacal sample were the reference categories. The aminoglycoside/aminocyclitol endpoint is not shown because the county-adjusted estimates were statistically unstable and were evaluated separately using bias-reduced sensitivity analyses. The x-axis is shown on a logarithmic scale, and the dashed vertical line indicates an odds ratio of 1.

Compared with breeder chickens (reference category), broiler chickens had significantly higher odds of resistance to beta-lactams (aOR = 4.56, P = 0.002), amphenicols (aOR = 14.96, P < 0.001), and fluoroquinolones (aOR = 6.34, P = 0.001). Broiler turkeys also exhibited increased odds of resistance to beta-lactams (aOR = 2.36, P = 0.008), tetracyclines (aOR = 2.38, P = 0.005), amphenicols (aOR = 14.45, P < 0.001), and fluoroquinolones (aOR = 2.57, P = 0.012). Resistance to amphenicols was elevated across nearly all production types, whereas, compared with breeder chickens, polymyxin resistance was significantly lower in broiler turkey isolates (aOR = 0.20, P < 0.001) and turkey breeder isolates (aOR = 0.09, P < 0.001). Folate pathway inhibitor resistance was lower in broiler turkeys (aOR = 0.22, P = 0.001). For egg-layer isolates, the estimated odds ratio for amphenicol resistance was 3.27 (95% CI: 1.00–10.69, P = 0.050), while for turkey breeder isolates the estimated odds ratio for folate pathway inhibitor resistance was 4.67 (95% CI: 0.84–25.96, P = 0.078). Sample origin (respiratory versus cloacal) was not significantly associated with resistance for any antimicrobial class.

The aminoglycoside/aminocyclitol-resistance endpoint required additional sensitivity analysis because the primary mixed-effects model produced exceptionally large and imprecise odds-ratio estimates (Supplementary Table S2). In the Firth bias-reduced logistic regression including poultry production type and sample type, the odds of aminoglycoside/aminocyclitol resistance relative to breeder chickens were higher for broiler chickens (OR = 6.90, 95% CI: 3.93–12.12), broiler turkeys (OR = 4.63, 95% CI: 2.87–7.47), and egg layers (OR = 2.78, 95% CI: 1.61–4.79), while the estimate for turkey breeder isolates was OR = 2.42 (95% CI: 1.42–4.15). Respiratory sample origin was not associated with aminoglycoside/aminocyclitol resistance (OR = 0.91, 95% CI: 0.67–1.24). However, additionally adjusting the Firth model for county caused several production-type estimates to change by orders of magnitude, demonstrating strong model dependence associated with the limited geographic overlap among production categories. Therefore, no county-adjusted aminoglycoside odds ratio was retained or interpreted as a stable production-type effect in the primary inferential results.

### Association between poultry type, sample type, and overall antimicrobial resistance burden

To complement the class-specific logistic regression analyses, overall resistance burden was evaluated as the number of antimicrobial class/group endpoints to which each isolate was resistant. An initial Poisson mixed-effects model showed substantial underdispersion (Pearson χ²/df = 0.46), indicating lower variability than expected under a standard Poisson distribution. A Conway–Maxwell–Poisson mixed-effects model was therefore fitted to accommodate the observed underdispersion and was retained for inference because it provided substantially improved model fit (AIC = 3156.4 vs. 3356.4). County was included as a random intercept, with poultry production type and sample type included as fixed effects (Table 5; Fig. 5).

**Table 5:** Incidence rate ratios (IRRs) for the number of resistant antimicrobial class/group endpoints per isolate estimated using a Conway-Maxwell-Poisson mixed-effects regression model^a^.

Predictor	IRR	Lower 95% CI	Upper 95% CI	P-value
Broiler chicken	1.63	1.38	1.93	<0.001
Broiler turkey	1.33	1.20	1.47	<0.001
Egg layer	1.24	1.04	1.48	0.015
Turkey breeder	0.98	0.87	1.10	0.681
Respiratory sample	1.00	0.96	1.05	0.931

a Counties were included as a random intercept, and poultry type and sample type were included as fixed effects. Breeder chickens and cloacal samples served as the reference categories. The Conway-Maxwell-Poisson model was retained because diagnostics indicated underdispersion in the standard Poisson model and substantially improved model fit (AIC = 3156.4 vs. 3356.4). CI = confidence interval.

**Fig. 5:**
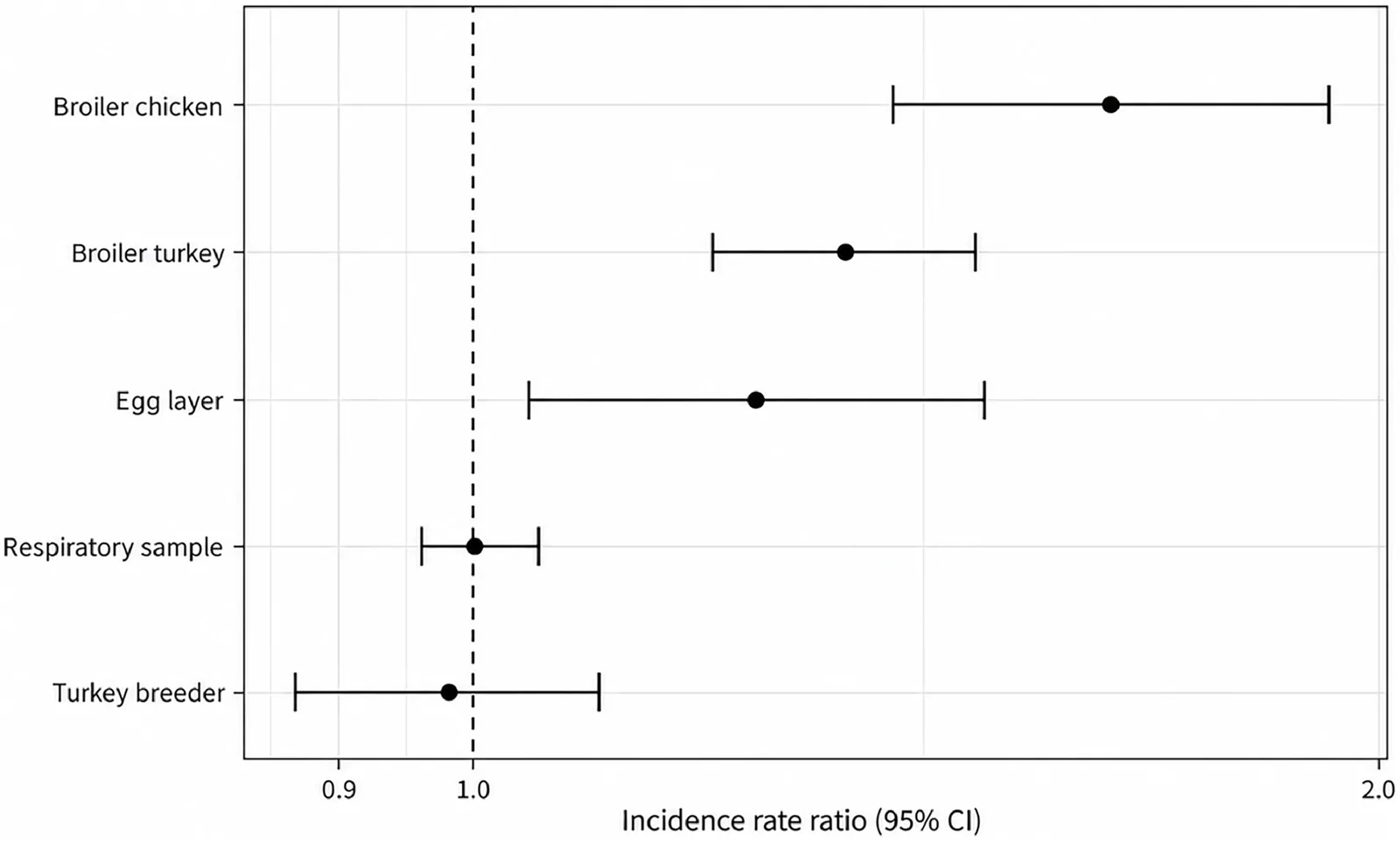
Association between poultry production type, sample type, and the number of resistant antimicrobial class/group endpoints per isolate estimated using a Conway–Maxwell–Poisson mixed-effects regression model with county included as a random intercept. Points represent incidence rate ratios and horizontal bars indicate 95% confidence intervals. Breeder chicken and cloacal sample were the reference categories. The dashed vertical line indicates an incidence rate ratio of 1.

The final Conway–Maxwell–Poisson model showed that the expected number of resistant antimicrobial class/group endpoints per isolate differed among poultry production types. Relative to breeder chickens, isolates from broiler chickens exhibited the highest multidrug resistance burden, with a 63% greater expected number of resistant antimicrobial classes (IRR = 1.63, 95% CI: 1.38–1.93, P < 0.001). Broiler turkey and egg-layer isolates also showed significantly higher resistance burdens, with 33% (IRR = 1.33, 95% CI: 1.20–1.47, P < 0.001) and 24% (IRR = 1.24, 95% CI: 1.04–1.48, P = 0.015) more resistant classes, respectively. In contrast, turkey breeder isolates did not differ significantly from breeder chickens (IRR = 0.98, 95% CI: 0.87–1.10, P = 0.681). Sample type was not associated with the number of resistant antimicrobial classes (IRR = 1.00, 95% CI: 0.96–1.05, P = 0.931).

## Discussion

The principal descriptive finding of this study was that phenotypic AMR patterns differed among *E. coli* isolates obtained from the evaluated poultry production categories. Broiler chicken and broiler turkey isolates showed particularly high MDR burdens, whereas other production categories exhibited different class-level resistance profiles. These observations support the value of examining poultry production categories separately rather than assuming a homogeneous poultry-wide resistance pattern. However, because several production categories were sampled from largely distinct sets of counties, the observed differences cannot be attributed exclusively to production type and may also reflect geographic variation (Grobbel et al., 2022; Sevilla-Navarro et al., 2022).

Notably, ceftriaxone resistance did not follow the overall production-type ranking observed for MDR: breeder chicken isolates showed the highest ceftriaxone resistance prevalence (38.8%) despite having the lowest overall MDR burden. This agent-specific pattern illustrates that production-category differences were not uniform across antimicrobials and should not be interpreted as a general increase in resistance across all agents in broiler systems. The present phenotypic dataset does not permit determination of the mechanisms underlying this ceftriaxone-specific pattern.

The use of *E. coli* isolates from clinically healthy poultry flocks as indicator organisms is well established in European surveillance programs, and our findings align with this framework. Harmonized EU monitoring relies on commensal *E. coli* to monitor resistance selection pressure and enable AMR comparisons across sectors and countries over time. The present cross-sectional dataset provides a descriptive comparison of AMR patterns among the sampled poultry categories. The observed heterogeneity indicates that poultry-level aggregation may conceal differences among production categories; however, the present sampling design was not intended to estimate national prevalence or temporal trends. Consequently, the findings should be considered complementary to, rather than equivalent to, structured national AMR surveillance data (Hesp et al., 2019; European Food Safety Authority (EFSA) and European Centre for Disease Prevention and Control (ECDC), 2023).

The most pronounced pattern was the high MDR burden observed in the broiler production categories. Broiler chicken isolates showed the highest observed MDR prevalence and elevated resistance estimates for several antimicrobial endpoints, while broiler turkey isolates also showed comparatively high resistance burdens. Because production type and county were incompletely overlapping, these model-based associations should not be interpreted as independent causal effects of production system; rather, they describe differences among the sampled production-category populations after the available county-level adjustment. This is biologically plausible because poultry systems differ in production duration, flock turnover, management intensity, and antimicrobial exposure, all of which influence AMR selection dynamics (Varga et al., 2019; Kerek et al., 2024). Cross-study comparisons of MDR prevalence should nevertheless be interpreted cautiously because MDR estimates depend on the antimicrobial agents and classes represented in the testing panel. In the present study, MDR was operationally determined from seven class/group endpoints; studies using broader or different antimicrobial panels may therefore yield systematically different MDR estimates even in otherwise comparable isolate populations.

Recent studies show that resistance patterns in broilers vary with age and sampling context, while layer and breeder systems exhibit distinct AMR and resistance-gene profiles. Together, these findings indicate that resistance selection in poultry may contribute to system-specific pressures rather than a uniform “poultry effect” (Caucci et al., 2019; Mbatidde et al., 2024; Liu et al., 2025).

Crude comparisons showed that turkey isolates carried higher resistance to amphenicols, fluoroquinolones, and tetracyclines, while folate pathway inhibitor resistance was already uniformly high. This suggests that chicken-turkey differences reflect shifts in class-specific resistance profiles rather than overall burden. Such patterns are consistent with comparative studies demonstrating compound- and system-specific divergence in AMR between broilers and turkeys (Varga et al., 2019). In Germany, for example, harmonized farm-level sampling has shown differences in multiple resistance endpoints between broiler and turkey *E. coli* populations. Similarly, studies from Spain and other settings have demonstrated that *E. coli* AMR patterns vary substantially by production environment, farming type, and antimicrobial context (Grobbel et al., 2022; Sevilla-Navarro et al., 2022; Khong et al., 2023; Deng et al., 2024; Monroy et al., 2025).

The chicken-turkey distinction may also extend along the food chain, as retail studies show that turkey meat can carry a distinct multidrug-resistant Enterobacteriaceae profile compared with chicken (Díaz-Jiménez et al., 2020). Occupational exposure studies further suggest alignment of AMR patterns among poultry, workers, and slaughterhouse environments, supporting shared exposure pathways (van den Bogaard et al., 2001). Although this was not a transmission study, relatively high MDR prevalence was observed among both broiler turkey and turkey breeder isolates in the present dataset.

The seven-cluster solution identified broad antimicrobial resistance-pattern groups and illustrated substantial heterogeneity in class-level co-resistance patterns, with broiler chicken and broiler turkey isolates distributed across multiple high-resistance profiles rather than a single dominant pattern (Hesp et al., 2021; Mesa-Varona et al., 2021). Several clusters were characterized by extensive multidrug resistance affecting five or more antimicrobial classes, whereas others were dominated by resistance to a smaller subset of antimicrobial classes. Broiler chicken and broiler turkey isolates were disproportionately represented in the largest multidrug-resistant clusters, suggesting that highly resistant phenotypes were concentrated within these production systems. However, resistance phenotypes were not exclusive to individual poultry types, indicating that similar resistance patterns occur across multiple production systems. The clustering analysis therefore complements the regression models by highlighting the complexity and heterogeneity of resistance-pattern structure beyond individual antimicrobial classes.

From a surveillance perspective, the observed differences suggest that stratification by poultry species and production category may provide information that is not apparent from aggregated poultry-level summaries. Nevertheless, confirmation in more geographically balanced sampling frameworks with retained farm- and flock-level identifiers is required before production-specific differences can be separated reliably from geographic and farm-level effects. Future surveillance designs incorporating such stratification may therefore help clarify whether production category represents an independently informative epidemiological factor (Davis et al., 2018; Caucci et al., 2019; Mesa-Varona et al., 2021; Mbatidde et al., 2024).

Several limitations should be considered when interpreting the results of this study. First, differences in MDR prevalence between this study and other surveillance programs should be interpreted cautiously because antimicrobial susceptibility panels vary among monitoring systems. In particular, the MDR definition used here was applied to seven antimicrobial classes, which may limit direct comparability with studies based on broader testing panels. An additional limitation is that farm- and flock-level identifiers were not retained after irreversible anonymization of the original field records. Because multiple isolates may have originated from the same farm or flock and this dependence could not be modeled, the confidence intervals and P values from the class-specific regression models should be regarded as potentially anti-conservative. If appreciable within-farm or within-flock correlation was present, confidence intervals may be narrower and P values smaller than would be obtained from a fully specified hierarchical model. Accordingly, these analyses represent isolate-level associations and not farm- or flock-level effect estimates.

Sensitivity to geographic model specification was not uniform across antimicrobial endpoints. County-fixed-effect Firth analyses produced broadly concordant directions for beta-lactam and polymyxin resistance and for several amphenicol and fluoroquinolone contrasts, but selected tetracycline, amphenicol, and fluoroquinolone estimates changed materially in magnitude or statistical classification. Aminoglycoside/aminocyclitol and folate-pathway inhibitor estimates were particularly unstable and imprecise. Accordingly, the class-specific regression coefficients should be interpreted primarily as associations within the observed sampling structure rather than as precise production-type effects independent of geography. The same structural limitation applies to the resistance-count model, whose IRRs likewise cannot be interpreted as production-system effects independent of geographic composition.

The collection of isolates was not based on nationally representative, slaughter-level sampling and should therefore be interpreted as a structured cross-sectional dataset rather than a national prevalence estimate. In addition, the analysis was limited to phenotypic data and lacked genomic, plasmid, and antimicrobial-use information, restricting mechanistic interpretation. Imipenem was retained within the beta-lactam framework but should be interpreted cautiously because it is not used in poultry production, and carbapenem resistance was not central to the stewardship context of this study.

Finally, the uneven geographic distribution of poultry production types may have influenced the observed associations. Although county was included as a random effect, production types occurred in largely distinct counties, making it difficult to fully separate production-type effects from geographic influences. Because farm-level location data were unavailable, within-county variation could not be assessed. Future studies with greater geographic overlap among production types and more detailed farm-level metadata are needed to better distinguish the effects of production system and location on antimicrobial resistance patterns. Accordingly, the adjusted production-type coefficients should be interpreted as associations within the observed sampling structure and not as estimates of production-type effects independent of geography.

Despite these limitations, the dataset includes a large number of phenotypically characterized *E. coli* isolates from clinically healthy chicken and turkey flocks and enables their analysis within a common laboratory and analytical framework. A particular strength is the inclusion of several poultry production categories, allowing descriptive comparison of resistance patterns that would otherwise be obscured by simple chicken-versus-turkey or poultry-wide aggregation (Grobbel et al., 2022; Kim et al., 2022).

Phenotypic antimicrobial resistance and multidrug resistance were common among *E. coli* isolates obtained from clinically healthy poultry flocks in Hungary, with substantial differences observed among the sampled poultry production categories. Broiler chicken and broiler turkey isolates showed particularly high MDR burdens. Because production categories were unevenly distributed across counties and farm- or flock-level dependence could not be modeled, these differences cannot be attributed solely to production system. The findings nevertheless indicate that stratifying poultry AMR data by species and production category may reveal epidemiological heterogeneity that is obscured by poultry-wide aggregation. Future geographically balanced surveillance with retained farm- and flock-level metadata is needed to determine the independent contribution of production system to AMR patterns.

## Funding

The research was supported by project no. RRF-2.3.1-21-2022-00001, which has been implemented with the support provided by the Recovery and Resilience Facility (RRF), financed under the National Recovery Fund budget estimate, RRF-2.3.1-21 funding scheme.

## Institutional ethics

The Animal Welfare Committee of the University of Veterinary Medicine Budapest confirmed that the study did not constitute an animal experiment and issued a formal Certificate of Exemption. Sample collection formed part of routine veterinary diagnostic activities, and no experimental infection, treatment, or other study-specific animal intervention was performed.

## Data availability

The datasets generated and/or analyzed during the current study are available from the corresponding author upon reasonable request.

## Author contributions

Ádám Kerek contributed to the conception and design of the study, methodology, investigation, data curation, formal analysis, visualization, manuscript drafting and critical revision, and supervision. Levente Hunor Husz, Edit Szarka, Máté Hetyésy, Gergely Álmos Tornyos, and Melinda Nemes-Terényi contributed to investigation, data acquisition and curation, and critical revision of the manuscript. László Kovács contributed to interpretation of the data, resources, supervision, and critical revision of the manuscript. Ákos Jerzsele contributed to the conception and design of the study, methodology, interpretation of the data, supervision, funding acquisition, and critical revision of the manuscript. Csaba Varga contributed to methodology, formal analysis, interpretation of the data, supervision, and critical revision of the manuscript. Each author therefore made a substantial contribution to the conception or design of the work and/or to the acquisition, analysis, or interpretation of data; participated in drafting the manuscript or critically revising it for important intellectual content; reviewed and explicitly approved the exact final version of the manuscript; and agrees to be personally accountable for all aspects of the work, including ensuring that any questions related to its accuracy or integrity are appropriately investigated and resolved.

## Disclosures

The authors declare no competing interests.

## Footnotes

Supplementary material associated with this article can be found, in the online version, at doi:10.1016/j.psj.2026.107750.
